# Effects of dietary gelatin hydrolysates on bone mineral density in magnesium-deficient rats

**DOI:** 10.1186/s12891-017-1745-4

**Published:** 2017-09-05

**Authors:** Teruyuki Noma, Satoshi Takasugi, Miho Shioyama, Taketo Yamaji, Hiroyuki Itou, Yoshio Suzuki, Keishoku Sakuraba, Keisuke Sawaki

**Affiliations:** 1 Division of Research and Development, Food Science Research Laboratories, Meiji Co., Ltd., 540 Naruda, Odawara, Kanagawa 250-0862 Japan; 2 Graduate School of Health and Sports Science, Juntendo University, Chiba, Japan; 3 Graduate School of Medicine, Juntendo University, Tokyo, Japan

**Keywords:** Collagen, Magnesium deficiency, Bone mineral density, Cortical bone, Trabecular bone, Rats, Peptide

## Abstract

**Background:**

The major types of commercially available gelatin hydrolysates are prepared from mammals or fish. Dietary gelatin hydrolysates from mammals were reported to improve bone mineral density (BMD) in some animal models. In contrast, there is limited study showing the effects of dietary gelatin hydrolysates from fish on BMD. The quantity and structure of peptides in the plasma after oral administration of gelatin hydrolysates depend on the gelatin source, which suggests that the biological activity of gelatin hydrolysates depend on the gelatin source. This study examined the effects of fish-derived gelatin hydrolysate (FGH) or porcine-derived gelatin hydrolysate (PGH) intake on BMD and intrinsic biomechanical properties in magnesium (Mg)-deficient rats as a model showing the decrease in both BMD and intrinsic biomechanical properties.

**Methods:**

Four-week-old male Wistar rats were assigned into four groups: a normal group was fed a normal diet (48 mg Mg/100 g diet), a Mg-deficient (MgD) group was fed a MgD diet (7 mg Mg/100 g diet), a FGH group was fed a MgD + FGH diet (5% FGH), and a PGH group was fed a MgD + PGH diet (5% PGH) for 8 weeks. At the end of the study, BMD and intrinsic biomechanical properties of the femur were measured.

**Results:**

The MgD group showed significantly lower Young’s modulus, an intrinsic biomechanical property, and trabecular BMD of the femur than the normal group; however, the MgD diet did not affect cortical BMD and cortical thickness. Both the FGH and the PGH groups showed significantly higher cortical thickness and ultimate displacement of the femur than the normal group, but neither type of gelatin hydrolysate affected Young’s modulus. Furthermore, the FGH group, but not the PGH group, showed significantly higher trabecular BMD than the MgD group.

**Conclusions:**

This study indicates that FGH and PGH increase cortical thickness but only FGH prevents the decrease in trabecular BMD seen in Mg-deficient rats, while neither type of gelatin hydrolysate affect intrinsic biomechanical properties.

## Background

Osteoporosis is a multifactorial bone disease, featured by low bone mineral density and microarchitectural deterioration of bone tissue, resulting in loss of mechanical strength and increased risk of fractures [1]. While calcium is the most well-known mineral for the prevention of osteoporosis, other minerals such as zinc, iron, and magnesium (Mg) also play an important role in bone metabolism. Magnesium is one of the nutrients most likely to be consumed at levels below the recommended daily allowance (RDA) [2]. Some cross-sectional studies have demonstrated that dietary Mg was positively correlated with bone mineral density (BMD) in elderly subjects [3] and middle-aged women [4], and a longitudinal study have also showed that a greater intake of Mg was associated with less of a decline in BMD in elderly subjects [3]. In rats, Mg deficiency was reported to reduce both BMD [5] and intrinsic biomechanical properties [6].

Collagen is a major component of connective tissues, such as bone, dermis, cartilage and tendons. Gelatin, a denatured collagen, is produced mainly from swine, fish, and birds. Gelatin hydrolysate is produced by the hydrolysis of gelatin, and the major types of commercially available gelatin hydrolysates are prepared from swine or fish. Some researchers reported that dietary gelatin or gelatin hydrolysates from mammals improve BMD in ovariectomized mice [7], growing rats [8], calcium-deficient rats [8], and low protein-fed rats [9]. In contrast, there is limited study showing the effects of dietary gelatin hydrolysates from fish on BMD [10].

Hydroxyproline (Hyp) is a major component of collagen. In vitro studies have shown that Hyp-containing peptides or gelatin hydrolysate-derived peptides have biological activity, including chemotactic activity for neutrophils, fibroblasts, [11, 12] and monocytes [13], as well as inhibitory effects on the angiotensin-converting enzyme [14, 15]. Ohara et al. [16] compared the structure and amount of Hyp-containing peptides in human plasma after peroral administration of fish-derived gelatin hydrolysates (FGH) or porcine skin-derived gelatin hydrolysates (PGH), and showed that the structure and amount of peptides in human plasma after peroral administration of gelatin hydrolysates depend on the gelatin source, and that alanine- or glycine-containing peptides were detected only in the human administered with FGH. These facts suggest that the biological activity of gelatin hydrolysates depend on the gelatin source.

This study aimed to investigate the effects of FGH and PGH intake on BMD and intrinsic biomechanical properties in Mg-deficient (MgD) rats as a model showing the decrease in both BMD and intrinsic biomechanical properties.

## Methods

### Diets

FGH and PGH were kindly provided by Nitta Gelatin (Osaka, Japan). Mean molecular weight of these gelatin hydrolysates was 5000 Da. We employed AIN-76 as the normal diet (48 mg Mg/100 g diet). The MgD diet contained 7 mg Mg/100 g diet. For MgD + FGH and MgD + PGH diets, the 5% casein in the MgD diet was replaced with FGH and PGH, respectively. The composition of each diet and analysis values of calcium, phosphorus, and Mg are detailed in Table 1.

**Table 1:** Composition of the experimental diets

Ingredients (%)	Normal	MgD	FGH	PGH
Casein	20.0	20.0	15.0	15.0
Fish scale gelatin hydrolysate	0.0	0.0	5.0	0.0
Porcine skin gelatin hydrolysate	0.0	0.0	0.0	5.0
DL-methionine	0.3	0.3	0.3	0.3
Corn starch	15.0	15.0	15.0	15.0
Sucrose	50.0	50.0	50.0	50.0
Corn oil	5.0	5.0	5.0	5.0
Cellulose powder	5.0	5.0	5.0	5.0
AIN-76 mineral premix	3.5	0.0	0.0	0.0
AIN-76 mineral premix without magnesium	0.0	3.5	3.5	3.5
AIN-76 vitamin mix	1.0	1.0	1.0	1.0
Choline bitartrate	0.2	0.2	0.2	0.2
Analysis values (mg/100 g)				
Calcium	533	530	524	521
Phosphorus	553	563	519	519
Magnesium	48	7	7	7

### Animals

This research was approved by the Institutional Animal Care and Use Committee (IACUC) board of the Meiji Co., Ltd. (Ethics approval code: No. 2015_3871_0088). Twenty-four, 3-week-old male Wistar rats (Japan SLC, Inc.,Shizuoka, Japan) were reared according to the Meiji Co., Ltd. ethics committee guidelines on animal use. The animals were housed in individual stainless steel cages in an environmentally controlled room (21 ± 2 °C, 55 ± 15% humidity, 12-h light/dark cycle). After 4 days of adaptation, animals were assigned into four weight-matched groups of six rats each: a normal group, an MgD group, a FGH group, and a PGH group. All groups were fed their respective experimental diets and ultraviolet sterilized water ad libitum for 8 weeks. Food intake and body weight were measured weekly. Food efficiency was calculated using the following formula:

$$ \mathrm{Food}\  \mathrm{efficiency}=\mathrm{body}\  \mathrm{weight}\  \mathrm{gain}/\mathrm{food}\  \mathrm{intake}. $$

At the end of the experimental period, we obtained blood samples from the abdominal aorta under anesthesia with a medetomidine-midazolam-butorphanol mixture [17]. All rats were euthanized by exsanguination through the aorta under the anesthesia. The serum samples were separated by centrifugation at 3000×g for 15 min at 4 °C and preserved at −80 °C until analysis. The both sides of femurs were excised after euthanasia. The left femurs were wrapped in saline-wet gauze and stored at −20 °C until mechanical testing as previously described [18]. The right femurs were preserved in a 70% solution of ethanol (Wako Pure Chemical Industries, Osaka, Japan) for X-ray computed tomography (CT) analysis.

### Bone parameters by X-ray CT analysis

The whole right femur was scanned using the experimental animal CT system (LaTheta LCT-100 M; ALOKA, Tokyo, Japan). Contiguous 1.0-mm slices of the whole femur were employed for quantitative measurement. Total, cortical, and trabecular BMDs, and cortical thickness (Ct.Th) of the whole femur were measured by LaTheta software (Version 1.31). Ct.Th of the whole femur was calculated as the mean of all slices. Ct.Th of each slice was calculated using the following formula:

$$ \mathrm{Ct}.\mathrm{Th}=\mathrm{cortical}\kern0.3em \mathrm{area}/\mathrm{cortical}\kern0.3em \mathrm{center}\kern0.3em \mathrm{line}\kern0.3em \mathrm{length}. $$

### Mechanical testing

We performed a three-point bending test by a mechanical testing system (Bone Strength Tester, model TK-252C; Muromachi Kikai, Tokyo, Japan) on the left femur according to the modified methods previously described [19, 20]. After placing the femur in a 37 °C saline bath, it was set on a supporter with two loading points 14 mm apart (the span of the specimen: L). A vertical breaking load was applied to the midpoint between the lower supports by the crosshead at a constant speed (2.5 mm/min) until failure occurred. The capacity of the load cell in this test system is 500 N. The work to failure, stiffness, ultimate displacement (d), and ultimate force (F) of the femur were calculated from the load–deformation curve. After failure, the cross-sectional area of the femur was calculated as a hollow ellipse [18, 21, 22]. The major and minor internal and external axes of the cross-sectional area were measured by digital caliper (DT-200; Niigata Seiki, Niigata, Japan). Ultimate stress and Young’s modulus were calculated as follows:

$$ \mathrm{Ultimate}\kern0.3em \mathrm{s}\mathrm{tress}=8\mathrm{bFL}/\left({\mathrm{a}\mathrm{b}}^3-{\mathrm{a}}^{\prime }{\mathrm{b}}^{\prime 3}\right)\pi {\mathrm{Young}}^{\prime}\mathrm{s}\kern0.3em \mathrm{modulus}=4{\mathrm{FL}}^3/3\mathrm{d}\left({\mathrm{a}\mathrm{b}}^3-{\mathrm{a}}^{\prime }{\mathrm{b}}^{\prime 3}\right)\pi $$

where a and b are the major and minor external axes, respectively, and a’ and b’ are the major and minor internal axes, respectively. The work to failure, stiffness, ultimate displacement, and ultimate force represent extrinsic biomechanical properties [23]. Ultimate stress and Young’s modulus represent intrinsic biomechanical properties. The work to failure reflects the absorbed energy of specimen until failure occurred. Increased brittleness reduces the work to failure. The stiffness reflects the resistance to elastic deformation, i.e., the structural rigidity of bone. The reciprocal of the ultimate displacement can estimate brittleness [23]. The ultimate force characterizes the strength of a bone.

### Biochemical analysis

Serum levels of total osteocalcin (OC) were assayed using the Rat Gla/Glu-Osteocalcin High Sensitive EIA Set (Takara Bio Inc., Shiga, Japan). Total OC was calculated as the sum of carboxylated OC and undercarboxylated OC. Serum C-terminal cross-linked telopeptides of type I collagen (CTX) and tartrate-resistant acid phosphatase 5b (TRACP5b) were measured by the RatLaps EIA kit and RatTRAP Assay kit, respectively, both of which were manufactured by Immunodiagnostic Systems Nordic A/S (Herlev, Denmark). Resorption index was calculated as the ratio of CTX/TRACP5b [24]. Serum Mg levels and alkaline phosphatase (ALP) activity were colorimetrically assayed by commercial kits (Wako Pure Chemical Industries, Osaka, Japan).

### Statistics

Data are expressed as mean ± standard errors. We performed Bartlett’s test to determine the homogeneity of variances. Treatment effects were analyzed by one-way ANOVA followed by the Tukey-Kramer test (homogenous variances) or the Kruskal–Wallis test followed by the Steel–Dwass multiple comparison test (heterogenous variances). *P* values <0.05 were considered significant. Additionally, we presented the minimum sample sizes to detect group differences between the MgD group and the FGH groups for BMD and trabecular BMD in the present study. We employed the Ekuseru–Toukei 2015 software (Social Survey Research Information Co., Ltd., Tokyo, Japan) to perform all statistical analyses.

## Results

### Growth parameters

**Fig. 1:**
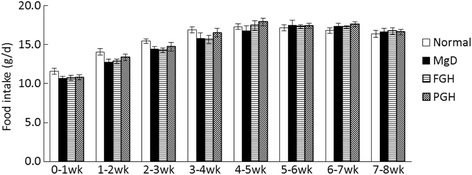
Food intake. Values are presented as mean ± SE. No significant difference among the groups was observed at all time points

**Fig. 2:**
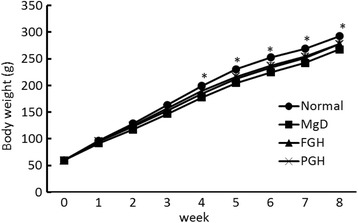
Body weight. Values are presented as mean ± SE. * *p* < 0.05 vs MgD

Food intake during the experimental period did not differ among the groups (Fig. 1, Table 2). There was no significant difference among the groups in body weight during the first 3 weeks of the experiment, but after that, body weight was significantly higher in the normal group than in the MgD group (Fig. 2). Final body weight and food efficiency were significantly lower in the MgD group than in the normal group.

**Table 2:** Growth parameters

	Normal	MgD	FGH	PGH
Food intake (g/d)	15.9 ± 0.2	15.4 ± 0.4	15.4 ± 0.3	15.7 ± 0.3
Final body weight (g)	292 ± 5^a^	268 ± 7^b^	277 ± 5^a, b^	277 ± 6^a, b^
Food efficiency (g/g)	0.247 ± 0.005^a^	0.227 ± 0.002^b^	0.237 ± 0.003^a, b^	0.233 ± 0.002^a, b^

Values are presented as mean ± SE. Values with different superscript letters within rows are significantly different (*p* < 0.05)

### BMD and Ct.Th of the whole femur

The BMD of the femur was significantly lower in the MgD and PGH groups than the normal group and tended to be higher (*p* = 0.0559) in the FGH group than the MgD group (Table 3). Ct.Th was significantly higher in the FGH and PGH groups than the normal group, and cortical BMD was significantly higher in the FGH group than the normal group. Trabecular BMD was significantly lower in the MgD, FGH, and PGH groups than in the normal group, but was significantly higher in the FGH group than the MgD group. The minimum sample sizes for detecting the differences between the MgD and FGH groups in the trabecular BMD and BMD at α = 0.05 (two-sided) and (1-β) = 0.8, determined using an unpaired t-test, were 7 and 5, respectively, which suggests that the sample size of this study seems to be statistically valid.

**Table 3:** Bone mineral density (BMD) and cortical thickness (Ct.Th) of the whole femur

	Normal	MgD	FGH	PGH
BMD (mg/cm^3^)	656 ± 3^a^	629 ± 5^b^	649 ± 7^a, b^	633 ± 6^b^
Cortical BMD (mg/cm^3^)	1058 ± 4^a^	1062 ± 4^a, b^	1073 ± 4^b^	1070 ± 2^a, b^
Trabecular BMD (mg/cm^3^)	393 ± 3^a^	358 ± 5^c^	375 ± 4^b^	361 ± 5^b, c^
Ct.Th (μm)	415 ± 2^a^	423 ± 4^a, b^	433 ± 6^b^	433 ± 4^b^

Values are presented as mean ± SE. Values with different superscript letters within rows are significantly different (*p* < 0.05)

### Mechanical testing

For the intrinsic biomechanical properties, there were no significant differences in the ultimate stress among the groups (Table 4). Young’s modulus was significantly lower in the MgD, FGH, and PGH groups than in the normal group.

**Table 4:** Intrinsic and extrinsic biomechanical properties of the femur by three-point bending test

	Normal	MgD	FGH	PGH
Intrinsic biomechanical properties				
Ultimate stress (MPa)	141 ± 4	134 ± 6	129 ± 4	132 ± 1
Young’s modulus (MPa)	1555 ± 68^a^	1250 ± 71^b^	1168 ± 66^b^	1138 ± 53^b^
Extrinsic biomechanical properties				
Ultimate force (N)	108 ± 3	115 ± 4	113 ± 3	122 ± 5
Ultimate displacement (mm)	1.00 ± 0.02^a^	1.14 ± 0.03^a, b^	1.18 ± 0.05^b^	1.23 ± 0.06^b^
Work to failure (mJ)	62.6 ± 3.0	66.3 ± 4.3	65.4 ± 2.1	76.2 ± 5.2
Stiffness (N/mm)	81.4 ± 1.8	85.3 ± 2.6	80.4 ± 3.1	87.1 ± 1.9

Values are presented as mean ± SE. Values with different superscript letters within rows are significantly different (*p* < 0.05)

For the extrinsic biomechanical properties, there were no significant differences in the ultimate force, work to failure, and stiffness among the groups; however, ultimate force and work to failure tended to be higher (*p* = 0.0689, *p* = 0.0912, respectively) in the PGH group than the normal group. Ultimate displacement was significantly higher in the FGH and PGH groups than the normal group.

### Biochemical factors

Serum Mg levels were significantly lower in the MgD, FGH, and PGH groups than the normal group (Table 5). There were no significant differences in the serum total OC, ALP activity, TRACP5b, and CTX among the groups. The CTX/TRACP5b ratio was significantly higher in the MgD group than the normal group. There were no significant differences in the CTX/TRACP5b ratio between the normal group and the FGH and PGH groups.

**Table 5:** Biochemical factors

	Normal	MgD	FGH	PGH
Mg (mg/dL)	1.65 ± 0.05^a^	0.50 ± 0.02^b^	0.52 ± 0.06^b^	0.47 ± 0.03^b^
Total OC (ng/mL)	273 ± 8	306 ± 19	303 ± 8	308 ± 8
ALP activity (units/μL)	0.126 ± 0.007	0.113 ± 0.007	0.119 ± 0.004	0.110 ± 0.004
TRACP5b (U/L)	11.3 ± 0.3	9.7 ± 0.8	10.7 ± 1.0	11.4 ± 1.1
CTX (ng/mL)	37.3 ± 1.1	43.1 ± 3.6	37.3 ± 1.3	40.6 ± 1.6
CTX/TRACP5b ratio (% vs Normal)	100 ± 4^a^	139 ± 12 ^b^	109 ± 7 ^a, b^	113 ± 10^a, b^

Values are presented as mean ± SE. Values with different superscript letters within rows are significantly different (*p* < 0.05)

## Discussion

This study aimed to investigate the effects of FGH and PGH intake on BMD and intrinsic biomechanical properties in Mg-deficient rats. In the present study, a low Mg diet had an adverse effect on trabecular BMD but did not affect cortical BMD, which was consistent with previous reports [25, 26]. A low Mg diet of 10% of the nutritional requirement was reported to increase bone resorption, while osteoblast numbers were similar to those seen in normal rats [26]. In the present study, a low Mg diet of 15% of the nutritional requirement increased CTX/TRACP5b ratio, known to be a useful osteoclast activity marker, but did not affect serum total OC and ALP activity, which are bone formation markers. The low Mg diet decreased Young’s modulus, an intrinsic biomechanical property, which is consistent with previous data [6]. In contrast, a low Mg diet did not affect extrinsic biomechanical properties. Cortical components have a prominent role in bone strength [27], which may explain why a low Mg diet did not affect extrinsic biomechanical properties despite a decrease in trabecular BMD and intrinsic biomechanical properties. In contrast, some approximations were made in calculating bone geometry for mechanical properties, or there could be a measurement sensitivity issue with the whole bone mechanical testing, which may also explain this inconsistency.

In this study, both types of gelatin hydrolysates increased the Ct.Th and ultimate displacement, but did not prevent the decrease in the intrinsic biomechanical property. These results suggest that gelatin hydrolysates attenuate bone brittleness by increasing Ct.Th in MgD rats without affecting intrinsic biomechanical properties. Neither collagen hydrolysate prevented the decrease in serum Mg levels. Furthermore, a previous study showed that PGH intake increases the diameter of the cortical area, even in ovariectomized mice [7]. These data suggest that the beneficial effect of the gelatin hydrolysates on Ct.Th was independent of Mg status. In the present study, both gelatin hydrolysate groups showed a similar CTX/TRACP5b ratio as the normal group, suggesting an improvement of osteoclast activity. In addition, Kim et al. [28] reported that PGH decreases the CTX/TRACP5b ratio in ovariectomized rats, which is consistent with our results. In the present study, the improvement of osteoclast activity may be the cause of the increase in Ct.Th.

Interestingly, only the administration of FGH prevented the decrease in trabecular BMD. Some studies reported that some oligopeptides may affect cell function and regulate gene expression in vitro [29]. Ohara et al. [16] compared quantities and structures of Hyp-containing peptides in human blood after administration of FGH and PGH and showed that the quantities and structures of peptides in human blood after oral administration of gelatin hydrolysates depend on the gelatin source, and alanine- or glycine-containing peptides were detected only in the subjects receiving FGH. An epidemiological study indicated that the intake of several amino acids, including alanine and glycine, may be beneficial for bone health [30]. Therefore, alanine- and/or glycine-containing peptides, derived from FGH, might benefit bone metabolism. Further studies are necessary to clarify the mechanism by which FGH beneficially affects trabecular BMD.

Our study has several limitations. First, we were not able to clarify whether the effects of FGH and PGH intake on BMD can be observed in other models, because we investigated the effects only in MgD rats. Second, some approximations were made in calculating bone geometry for mechanical properties, which may cause the variation. Third, there could be a measurement sensitivity issue with the whole bone mechanical testing.

## Conclusions

The present study indicated that both FGH and PGH increase Ct.Th, but only FGH prevents the MgD-induced decrease in trabecular BMD seen in MgD rats, while neither type of gelatin hydrolysate affect intrinsic biomechanical properties.

## Abbreviations

ALP: Alkaline phosphatase
ANOVA: Analysis of variance
BMD: Bone mineral density
CT: Computed tomography
Ct.Th: Cortical thickness
CTX: C-terminal cross-linked telopeptides of type I collagen
FGH: Fish-derived gelatin hydrolysates
Hyp: Hydroxyproline
IACUC: Institutional Animal Care and Use Committee
Mg: Magnesium
MgD: Mg-deficient
OC: Osteocalcin
PGH: Porcine skin-derived gelatin hydrolysates
RDA: Recommended daily allowance
TRACP5b: Tartrate-resistant acid phosphatase 5b
