# Correction to: effects of dietary gelatin hydrolysates on bone mineral density in magnesium-deficient rats

**DOI:** 10.1186/s12891-017-1774-z

**Published:** 2017-10-24

**Authors:** Teruyuki Noma, Satoshi Takasugi, Miho Shioyama, Taketo Yamaji, Hiroyuki Itou, Yoshio Suzuki, Keishoku Sakuraba, Keisuke Sawaki

**Affiliations:** 1 Division of Research and Development, Food Science Research Laboratories, Meiji Co., Ltd., 540 Naruda, Odawara, Kanagawa 250-0862 Japan; 2 Graduate School of Health and Sports Science, Juntendo University, Chiba, Japan; 3 Graduate School of Medicine, Juntendo University, Tokyo, Japan

After the publication of this article [1] the authors pointed out to us that a formula was incorrectly shown due to an error during typesetting as:

$$ \mathrm{Ultimate}\  \mathrm{stress}=8\mathrm{bFL}/\left({\mathrm{a}\mathrm{b}}^3\hbox{--} {\mathrm{a}}^{\prime }{\mathrm{b}}^{\prime }3\right)\uppi $$

$$ {\mathrm{Young}}^{'}\mathrm{s}\ \mathrm{modulus}={4\mathrm{FL}}^3/3\mathrm{d}\left({\mathrm{a}\mathrm{b}}^3\hbox{--} {\mathrm{a}}^{\prime }{\mathrm{b}}^{\prime }3\right)\uppi $$

It should have been shown as:

$$ \mathrm{Ultimate}\  \mathrm{stress}=8\mathrm{bFL}/\left({\mathrm{a}\mathrm{b}}^3\hbox{--} {\mathrm{a}}^{\prime }{{\mathrm{b}}^{\prime}}^3\right)\uppi $$

$$ {\mathrm{Young}}^{'}\mathrm{s}\ \mathrm{modulus}={4\mathrm{FL}}^3/3\mathrm{d}\left({\mathrm{a}\mathrm{b}}^3\hbox{--} {\mathrm{a}}^{\prime }{{\mathrm{b}}^{\prime}}^3\right)\uppi $$

BMC apologise for this error. The original article was corrected.
